# Harnessing the Membrane Translocation Properties of AB Toxins for Therapeutic Applications

**DOI:** 10.3390/toxins13010036

**Published:** 2021-01-06

**Authors:** Numa Piot, F. Gisou van der Goot, Oksana A. Sergeeva

**Affiliations:** Global Health Institute, School of Life Sciences, EPFL, 1015 Lausanne, Switzerland; numa.piot@epfl.ch (N.P.); gisou.vandergoot@epfl.ch (F.G.v.d.G.)

**Keywords:** botulinum toxin, anthrax toxin, cholera toxin, membrane translocation, endocytosis, therapeutic applications, drug delivery

## Abstract

Over the last few decades, proteins and peptides have become increasingly more common as FDA-approved drugs, despite their inefficient delivery due to their inability to cross the plasma membrane. In this context, bacterial two-component systems, termed AB toxins, use various protein-based membrane translocation mechanisms to deliver toxins into cells, and these mechanisms could provide new insights into the development of bio-based drug delivery systems. These toxins have great potential as therapies both because of their intrinsic properties as well as the modular characteristics of both subunits, which make them highly amenable to conjugation with various drug classes. This review focuses on the therapeutical approaches involving the internalization mechanisms of three representative AB toxins: botulinum toxin type A, anthrax toxin, and cholera toxin. We showcase several specific examples of the use of these toxins to develop new therapeutic strategies for numerous diseases and explain what makes these toxins promising tools in the development of drugs and drug delivery systems.

## 1. Introduction

The FDA is increasingly approving biological drugs. In 2018, these protein-based drugs made up 25% of FDA approvals and included antibodies, growth factors, hormones, and enzymes that target a broad range of diseases [1]. The market for such drugs is expected to increase over the next few years due to their interesting properties [2]. Compared to traditional small-molecule drugs, protein- or peptide-based drugs generally show high specificity, high efficacy and high selectivity, and allow the development of drugs for a broad range of targets, notably in cancer treatment [3]. The increasing market share for biologics is even more impressive considering the inability of most of these drugs to cross the cellular plasma membrane and reach the cytosol, making their delivery a huge challenge that currently hinders the field [4]. Indeed, tools that could improve the delivery of biologics, especially those that could be applied broadly, would see immediate application and greatly benefit the drug-delivery field. Furthermore, if delivery were sufficiently efficient, the range of potential drug targets would be drastically broadened due to the increased accessibility of intracellular proteins.

Fortunately, a naturally evolved system for delivering functional proteins into cells is provided by bacterial toxins, which have evolved to hijack cellular internalization mechanisms and developed membrane translocation devices for exactly this purpose. AB toxins are a family of bacterial toxins that include diphtheria toxin, cholera toxin, anthrax toxin, Shiga toxin, and botulinum toxin, among others [5,6,7,8,9]. They are named for their two components: an active part (A) that is responsible for the catalytic activity of the toxin, and a binding part (B) that is involved in binding to the cell membrane and escorting the A subunit to its destination. AB toxins have finely tuned their cellular entry to evade host defenses, providing hints and tools for protein-based drug development. In this review, we focus on how the internalization mechanisms of three AB toxins—botulinum toxin type A, anthrax toxin, and cholera toxin—can be used in different therapeutic approaches (Table 1). We decided to focus on these three toxins based on the strong modular potential of anthrax toxin, on the already approved use of botulinum toxin type A in several neurological disorders and on the wide variety of therapeutical applications of cholera toxin. The practical approaches presented in this review take advantage of both the intrinsic properties of the toxins as well as the modularity of both the A and B subunits, all aspects that can be further extended to other AB toxins.

## 2. Botulinum Toxin Type A

### 2.1. Botulinum Toxin Type A Internalization Mechanism

As part of the AB toxin family, botulinum neurotoxins consist of seven different toxin types (termed A–G) and are produced by the *Clostridium* family of bacteria [7]. Generally, the toxin reaches the bloodstream by using transcytosis to cross the epithelial layer of the lungs or the gastrointestinal tract [62]. This review will briefly depict the internalization mechanism of botulinum toxin type A (BoNT/A), which is the most-studied toxin subtype with the most FDA-approved therapeutical applications. For more detailed informations about this particular process, the readers are referred to previously published literature [7,63]

**Figure 1:**
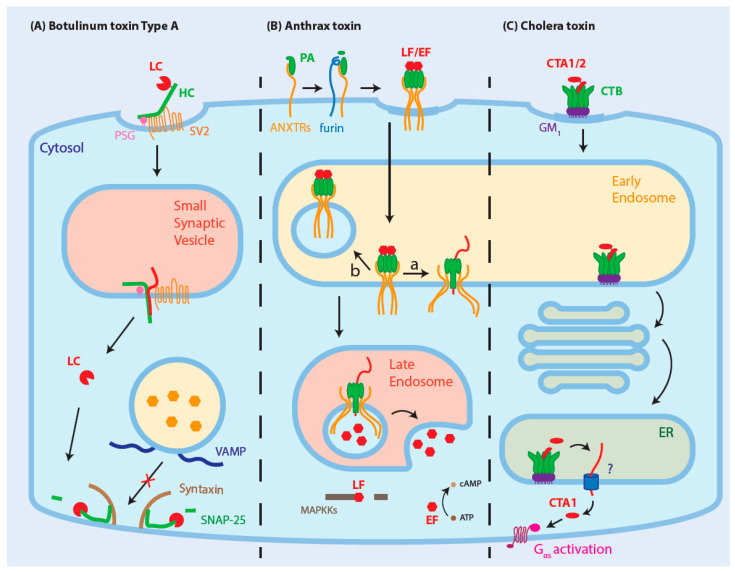
Internalization mechanisms of botulinum toxin type A, anthrax toxin, and cholera toxin. (**A**) Botulinum toxin binds to polysialogangliosides (PSGs) and then to synaptic vesicle protein 2 (SV2), which leads to the internalization of the toxin in small synaptic vesicles. The low pH induces a structural change of botulinum toxin heavy chain (HC) that leads to the unfolding of the light chain (LC) and its translocation through the membrane. Once in the cytosol, the disulfide bond between the HC and LC is reduced, and the LC refolds. LC cleaves SNAP-25 and impairs synaptic vesicle fusion. (**B**) Anthrax toxin binds to its receptors, CMG2 or TEM8, and is cleaved by a furin-family protease. In this form, PA oligomerizes and clusters in lipid rafts at the plasma membrane. The oligomeric form of PA recruits LF or EF. The receptor-PA complex is endocytosed and is targeted to early endosomes. While some PA pores start to form at the limiting membrane of endosomes (a), some are sorted in intraluminal vesicles (ILVs) and targeted to lysosomes (b). On the way to lysosomes, the PA oligomers undergo pH-dependent PA pore formation in the membrane of ILVs. The pores allow the translocation of unfolded LF through the membrane. These vesicles fuse with the limiting membrane of late endosomes and release their content in the cytosol, where LF cleaves MAPKKs and EF converts ATP into cAMP. (**C**) The cholera toxin B subunit binds in a pentameric form to the membrane on GM1 in lipid raft domains of the plasma membrane. CTA2 interacts with the pentamer and links the catalytically active CTA1 subunit via a disulfide bond. Once endocytosed in endosomes, the toxin is transported to the trans-Golgi network (TGN) and then to the endoplasmic reticulum (ER) using retro-translocation. The reductive environment of the ER frees CTA1 by breaking the disulfide bond, which is then translocated through the ER membrane using ERAD-associated mechanisms. In the cytosol, CTA1 constitutively activates Gαs, increasing cAMP levels.

BoNT/A is composed of a catalytic subunit, the 50-kDa light chain (LC), connected by a disulfide bridge to the binding subunit, a 100-kDa heavy chain (HC), responsible for the binding and translocation of the catalytic subunit into the cytosol (Figure 1A) [10]. The HC first recognizes polysialogangliosides (PSGs) at the nerve terminal and then stabilizes the binding by a high-affinity interaction with synaptic vesicle protein 2 (SV2) [11,19]. Endocytosis of BoNT/A targets it to small synaptic vesicles, which was shown to be enhanced by synaptic vesicle recycling induced by neuronal activity [20,24].

The exact membrane translocation mechanism remains unsolved, though experiments with lipid bilayer modeling this process were reviewed by Pirazzini et al. [64]. According to the models, the acidic environment of synaptic vesicles induces a structural change of the HC, converting it into a chaperone and an ion channel that unfolds LC and shuttles it across the membrane of the vesicle to the cytosol [64]. In the cytosol, LC refolds into a Zn^2+^-dependent metalloprotease that hydrolyses a specific peptide bond in SNAP-25, which is part of the cytosolic membrane fusion complex, termed SNARE [28,65,66]. This complex consists of three different proteins: SNAP-25, syntaxin, and vesicle-associated membrane protein (VAMP), the latter two being targets of the other botulinum toxin types [67,68]. The cleavage of SNAP-25 by BoNT/A in neurons inhibits the fusion of synaptic vesicles, and thus the release of neurotransmitters in the synapses, leading to paralysis.

While the half-life of the toxin in the bloodstream is approximately four hours, the lifetime of BoNT/A is drastically increased once it reaches the cytosol due to its high stability and resistance to proteasomal degradation [69,70]. The very long lifetime of the toxin explains how it can induce paralysis for up to 6 months in humans. Although botulinum toxin subtypes seem to internalize using the same pathway, they bind to different receptors with variable expression in the different neuronal cell types and their catalytic subunits target different proteins of the SNARE complex, inducing variations in the inhibition of synaptic vesicle fusion. These two aspects of botulinum toxin, aside from its intrinsic therapeutic properties, allow for the development of new therapeutic strategies for numerous diseases.

### 2.2. Botulinum Toxin Type A Therapeutic Applications

Under the name of Botox^®^, botulinum toxin is well known for its use in cosmetic treatments, as its effect on acetylcholine release by motoneurons at the neuromuscular junction leads to muscle relaxation. This is of great interest in muscle hyperactivation disorders. In this context, BoNT/A was first approved by the FDA in 1989 for the treatment of blepharospasm, a hyperactivation of the eyelid muscles that leads to repetitive and uncontrolled eyelid quivering. The long-term effect of the toxin, which can last for months, and the low diffusion rate in the extracellular fluid, which allows restricted and precise treatment in targeted body areas, make BoNT/A an invaluable therapeutic tool [30]. Since its initial approval, botulinum toxin has shown promise in the treatment of many other dystonic and spastic disorders which are associated with the dysfunction of the skeletal nervous system and voluntary body movements, such as cervical dystonia, laryngeal dystonia, or upper and lower limb spasticity [31]. BoNT/A also inhibits the autonomic nervous system, which controls unconscious muscular contractions, and was used in the treatment of several autonomic disorders, such as hyperhidrosis, hypersalivation, allergic rhinitis, as well as some urologic and gastrointestinal disorders characterized by hyperactivation of smooth muscles [71].

The development of therapeutic BoNT/A also showed a nociceptive effect that was first considered to be a consequence of muscle relaxation [72], though it was later shown that the reduction in pain was a direct effect of BoNT/A on the nociceptor system. This effect was induced by a combination of the inhibition of neuropeptides and the release of anti-inflammatory mediators with a decrease in the transport of pain sensors, such as TRPV1 and TRPA1, at the plasma membrane in a process that relies on SNAP-25 [7]. Furthermore, several publications showed a distant action of the toxin in CNS regions following localized peripheral injections, indicating that the nociceptive function of botulinum toxin might arise from retro and anterograde axonal transport, which is responsible for the movement of different organelles to and from the neuronal cell body, towards the central nervous system [73,74]. Indeed, BoNT/A has been FDA-approved for the treatment of chronic migraines and also showed efficacy in the treatment of chronic headaches and certain neuropathies, such as postherpetic neuralgia, post-traumatic neuralgia, and painful diabetic neuropathy [63].

The intrinsic properties of botulinum toxin have made it an effective therapeutic for many seemingly unrelated disorders, though the major therapeutic potential of BoNT/A lies in its modularity. For example, the seven currently recognized serotypes (A-G) of botulinum toxin each have several subtypes (A1, C2, etc.) [75]. All these toxins have the same general structure consisting of one catalytic domain (LC), one binding domain, and one translocation domain (HC), but they each have specific binding affinities for different receptors and distinctive cleavage sites on various targets [68,76]. This modularity is normally used by bacteria to target different neuronal membranes and induce various deleterious effects, though it has also been elegantly exploited by Rummel et al. They swapped the N-terminal and C-terminal domain of the HC of several botulinum toxin types and showed that these domains can modulate toxin affinity for unique neuronal membranes [77]. In this context, a botulinum toxin chimera was designed with the HC of BoNT/B (HC_B_), due to higher concentration of the HC_B_ receptor, Synaptotagmin II (SytII), over HC_A_ receptors for an increased uptake in synaptic vesicles, and the LC of BoNT/A (LC_A_), due to its longer lifetime in the cytosol compared to LC_B_. This chimera induced a prolonged neuromuscular paralysis in mice of 50 days, compared to 30 days when using the full-length BoNT/A [49].

Similarly, Wang et al. made a chimeric botulinum toxin to target and suppress the release of the pain signaling peptide, calcitonin gene-related peptide (CGRP), by sensory neurons. This distinctive specificity was achieved due to the properties of the three different chains of the chimera, which was composed of LC_E_ fused to a mutated inactive form of LC_A_ (mLC_A_), both connected to the HC_A_ that internalized the fused LCs in the cytosol [38]. In this chimera, internalization was achieved because sensory neurons express the HC_A_ receptor isoform SV2C, but not the HC_E_ receptor isoforms SV2A and B [19,78]. A long-lasting effect was due to the presence of a dileucine motif in mLC_A_ that plays a role in its protection from proteasomal degradation [79]. Finally, the strong inhibition of CGRP release is due to the LC_E_-induced cleavage of 26 amino acids from the C-terminal of SNAP-25, while LC_A_ cleaves only 9. Taken together, this LC_E_-mLC_A_-HC_A_ chimera showed strong nociceptive inhibition both in vitro in trigerminal ganglion neurons and in vivo in mice [38]. These two chimera examples perfectly illustrate how the modularity of the different types of botulinum toxin can affect their therapeutic applications.

Using the same strategy, fusion proteins of botulinum toxin with other proteins were created in order to modulate the targeted receptor and, thus, the targeted cell type. To develop a treatment for acromegaly, an endocrine disorder characterized by an increased secretion of pituitary growth hormone (GH), Somm et al. made a modified fusion construct of the GH-releasing hormone with the translocation domain of HC_D_ and the LC_D_, which cleaves one of the protein of the SNARE complex responsible for GH secretion, VAMP2 [50]. This construct decreased GH production and secretion in vivo, which reduced the body weight and body size of juvenile rats. Similarly, a study using a botulinum toxin fusion construct with wheat germ agglutinin inhibited insulin secretion in hamster pancreatic cells [51]. Together, these examples further illustrate the extraordinarily broad spectrum of therapeutic applications of AB toxins and how the properties of the bacterial toxins can be exploited to achieve a targeted therapeutic strategy.

## 3. Anthrax Toxin

### 3.1. Anthrax Toxin Internalization Mechanism

An important concern for animal health and human public safety in the context of bioterrorism, anthrax toxin is an AB toxin produced by the gram-positive spore-forming bacterium *Bacillus anthracis*. This toxin consists of a B subunit, protective antigen (PA), and two catalytic A subunits, lethal factor (LF) and edema factor (EF). PA is an 83-kDa protein that is responsible for the binding of the toxin to its main receptors, capillary morphogenesis 2 (CMG2) and tumor endothelial marker 8 (TEM8) [12,13]. LF is an 91-kDa matrix metalloprotease that cleaves the MAPKK family members, which impairs the associated signaling pathways and eventually leads to apoptosis, especially in macrophages [29,80]. EF is a calmodulin-dependent adenylyl cyclase that increases the cytosolic cAMP levels. This review briefly describes the internalization process of anthrax toxin and, for a more in-depth understanding of this mechanism, readers are oriented towards previously published reviews [6].

Initially in LF and EF internalization, extracellular PA binds to one of its receptors, CMG2 or TEM8, and then is cleaved by furin-family proteins (Figure 1B). This cleavage allows PA to oligomerize into heptamers or octamers, also called pre-pores [15,16,81], which can then recruit three or four LF or EF subunits, respectively, for internalization. On the cytosolic side, PA binding to the TEM8 or CMG2 receptor causes it to release from the actin cytoskeleton [82,83], allowing ubiquitination of the receptor, which triggers endocytosis of the receptor-anthrax toxins complex [82].

Anthrax toxin and its receptors are then targeted to early endosomes where they are sorted in endosomal intraluminal vesicles (ILVs) and trafficked through the endocytic pathway towards late endosomes [21]. On the way to late endosomes, the acidification of the microenvironment induces a conformational change in the PA pore [25], and this low pH is also required for the translocation of LF [26]. Pores can form at the limiting membrane of the endosomes, translocating LF or EF directly into the cytosol, though most pores form in the membrane of ILVs [21,84]. These pores allow the translocation of LF or EF to the lumen of ILVs and, by back-fusion of ILVs with the limiting membrane of late endosomes, LF or EF eventually reaches the cytosol [21]. In opposition to BoNT/A, evidence suggest that LF has a very short half-life in the cytosol and its long-term effect relies on its ability to remain dormant in ILVs which stochastically back-fuse with the membrane of endosomes over a long period of time [21].

### 3.2. Anthrax Toxin Therapeutic Applications

The therapeutic potential of anthrax lethal toxin was originally exploited in anti-cancer treatments due to its inhibitory effect on the MAPKK-associated pathway. Unlike normal cells, cancer cells usually rely on only a few dysregulated pathways to increase their growth, survival, or motility. Accordingly, some cancers, such as melanoma bearing the V600E BRAF mutation, mostly rely on the constitutively activated MAPK pathway for cell growth and survival, and anthrax toxin was shown to decrease both these processes in this particular cell line [32]. Similarly, anthrax lethal toxin was shown to reduce cell growth and tumor angiogenesis in renal cell carcinoma and to reduce cell motility and invasiveness in astrocytes by targeting the MAPK pathway [33].

Although anthrax lethal toxin showed interesting intrinsic anti-tumor properties, most of its potential in therapy relies on its modular properties, like its ability to translocate different non-native proteins, drugs, and other molecules. As mentioned previously, PA oligomers create a pore in endosomes, allowing LF to eventually reach the cytosol, suggesting that LF fusion proteins could go through the pore as well—as long as they can successfully unfold while passing through the pore and refold later in the cytosol. In the 1990s, the first attempts to fuse proteins to the N-terminus of the LF subunit were done to target proteins to the cytosol and confirm the potential of anthrax toxin as a delivery system. FP59, a fusion between the N-terminus of LF (LF_N_) with the ADP-ribosylation domains of Pseudomonas exotoxin A, was the first successful translocation of a foreign protein into the cytosol [39]. Shortly after, both catalytic domains of the Shiga and diphtheria toxins reached the cytosol when fused to LF_N_, further supporting that the N-terminal residues of LF were sufficient to translocate complicated polypeptide chains through the PA pore [40,41]. However, Blanke et al. later showed that a simple positively-charged polycationic peptide could replace LF_N_ for the delivery of diphtheria toxin to the cytosol [42].

Besides bacterial toxins, the LF_N_ delivery system was shown to be useful in other applications, such as the development of a potential HIV vaccine and the treatment of neurodegenerative diseases [43,44]. In a broader perspective, Rabideau et al. assessed the feasibility of translocation through the PA pore for many different cargo molecules, from short or cyclic peptides to small molecule drugs. They concluded that while non-canonical peptides and small-molecule drugs, such as doxorubicin, can be translocated, cyclic peptides and the small molecule docetaxel cannot, which they hypothesized was due to rigidity of the cargo [45]. These examples provide strong evidence that many different cargo proteins can be delivered to the cytosol both in vitro and in vivo using anthrax toxin, which can be used for the targeted delivery of vaccines, drugs, and other proteins.

Besides its ability to translocate different non-native cargos, another modular characteristic of PA lies in the specificity of the protease that processes it, thereby allowing it to oligomerize. In the last two decades, several groups focused on unraveling the best combinations of mutations in PA that would allow more targeted and less toxic tumor therapies. The two PA mutants, PA-L1 and PA-U2, were programmed to be specific for several cancer cell lines in vitro by changing the cleavage site from furin to matrix metalloproteases (MMPs) and urokinase plasminogen activator (uPA), respectively [52,53], which are overexpressed in many cancer types while not very abundant at the surface of normal cells. In particular, PA-U2 showed a strong anti-tumor activity and specificity when combined with FP59 in mice [54]. To make the tumor targeting more specific, PA-L1 and PA-U2 were mutated on their homo-oligomerization domain to render them complementary, making them even more specific to cancer cells expressing both proteases. This approach was shown to be efficient with different sets of PA mutants both in vitro and in vivo [85,86,87,88]. In addition to their use as anti-tumor drugs, the protease-specific PA mutants were also used in combination with radioactively labelled LF or LF_N_-β-lactamase fusion protein to develop methods of imaging plasma membrane protease activity in tumors or in cancer cell lines, respectively [89,90].

Using the potential of PA to internalize molecules, several research groups adapted this technology to allow cancer-specific receptors to bind and internalize PA-fusions specific for those receptors. Varughese et al. were the first to unravel the potential of this strategy by targeting FP59 to a c-Myc-specific 9E10 hybridoma cell line using a PA-c-Myc fusion protein [55]. McCluskey et al. used a similar approach containing a mutated PA (mPA) that cannot bind its natural receptors fused with a high-affinity Affibody, ZHER2, targeting the HER2 receptor [56]. They showed that both mPA-EGF and mPA-ZHER2 could deliver an LF_N_-fused diphtheria toxin catalytic domain (DTA) to kill several cancer cell lines depending on the presence of their respective receptors [56]. Based on these observations, PA can form pores and deliver cargos as long as the targeted receptor is able to internalize, broadening the number of potential targets at the cell surface of cancer cells.

Additionally, Loftis et al. used an mPA fused with the single-chain variable fragment (scFv) of an antibody to internalize and deliver LF_N_-DTA through EGFR or carcinoembryonic antigen, which could kill pancreatic cancer cells overexpressing the two receptors at the plasma membrane. For additional specificity towards their pancreatic cancer cell line, they made an LF-RRSP fusion protein which targets the Ras–ERK signaling pathway, crucial for many pancreatic cancer cells [57]. Similarly, Becker et al. used designed ankyrin repeat proteins (DARPins) fused to a PA-CMG2-based construct to specifically target transmembrane glycoprotein epithelial cell adhesion molecule (EpCAM) at the surface of cells. These engineered constructs were shown to target EpCAM-expressing cells with a high specificity and to deliver LF_N_-based constructs to the cytosol [58]. Overall, these engineered proteins show that both the A and B subunits of anthrax toxin have strong potential as a protein delivery system, and they open many new routes for investigating the development of therapeutics. However, the immunogenicity of anthrax toxin subunits, as illustrated by the use of PA in anthrax vaccines, for example, remain a challenge to address in its therapeutical applications [91].

## 4. Cholera Toxin

### 4.1. Cholera Toxin Internalization Mechanism

Cholera toxin (CT) is an AB toxin of the heat-labile enterotoxin family and is produced by the bacterium *Vibrio cholerae*. The functional B subunit is actually a 55-kDa pentameric ring of individual B subunits (CTB) that tightly bind to its glycolipid receptor, GM1. The A subunit consists of two parts: an 11-kDa catalytically active CTA1 subunit and a 18-kDa CTA2 subunit, whose role is to anchor CTA1 in the lumen of the B-pentameric ring [5,17,18]. CTA1 is an ADP-ribosyltransferase that constitutively activates the heterotrimeric G-protein, Gαs. CTA1 and CTA2 are connected through a flexible linker containing a disulfide bridge. For a more detailed description of the cholera toxin internalization process, readers are referred to the following reviews [5,14].

Once bound to its receptor, CT associates with the GM1- and cholesterol-rich lipid rafts at the plasma membrane, which are necessary for efficient endocytosis of the toxin (Figure 1C) [22]. Once endocytosed, the toxin reaches early endosomes where it is targeted to the trans-Golgi network (TGN) via retrograde transport [23]. From there, CT bypasses the Golgi stacks and directly reaches the reductive environment of the ER [92], wherein the disulfide bridge between CTA1 and CTA2 is reduced and protein disulfide isomerase finishes the separation of both CTA subunits [27,93]. CTA1 is then thought to spontaneously unfold at physiological temperature. At that stage, it is thought to mimic a misfolded protein leading to its recognition by the ER-associated degradation (ERAD)-dependent pathway and its retro-translocation into the cytosol [14]. The C-terminus of CTA1 contains a KDEL motif that is not necessary for endosome to ER retrograde transport, but it is thought to play a role in ER retention once CTA1 dissociates from CTA2 and CTB [94]. In the cytosol, the low number of lysines in CTA1 most likely protects it from ubiquitination and further degradation by the proteasome [95]. Its ADP-ribosyltransferase activity then activates Gαs, which in turn increases cAMP levels in the cell, impairing sodium uptake and increasing chloride extrusion. Eventually, this induces the secretion of water and leads to intense diarrhea [14].

### 4.2. Cholera Toxin Therapeutic Applications

CT has been known for decades to have immunogenic properties. As early as 1984, it was used as an adjuvant in mucosal vaccines, as it was able to trigger both a mucosal and systemic antibody response [34,35]. It was also shown that the CTA-induced toxicity could be avoided by triggering the immune response through the use of only CTB [96]. Besides co-injection of the CTB adjuvant with different antigens, the immune response could be improved by conjugating CTB with an antigen [96]. This improvement is likely due to the broad presence of GM1 in many immune cells (B cells, T cells, macrophages, dendritic cells), as well as in epithelial cells and neurons, which would increase the uptake of the antigen-conjugated CTB in those cells [97]. This strategy has been used for the development of mucosal vaccines against a wide range of bacteria, viruses, and parasites in mice, as reviewed in previous publications [59,98]. Additionally, several other groups used the non-toxic CTA2 subunit as a fusion protein, co-injected with CTB, to develop their mucosal vaccine [46,47]. For example, Tinker et al. developed a mucosal vaccine against West Nile Virus using domain III of the virus envelope conjugated to CTA2 and the CTB subunit. The fusion protein was shown to efficiently bind to the plasma membrane, internalize into the perinuclear region of Vero and DC2.4 dendritic cells in vitro, and induce an increased production of IgG and IgM in mice after several injections [48]. Although the immunogenic effect of CTB has been exploited as previously mentioned, one drawback of this immunogenicity lies in the production of neutralizing antibodies towards CTB, which can be an issue if used as a therapeutic or as a drug delivery system. This particular topic will be discussed further below.

In addition to immunogenicity, CT was also shown to have immunosuppressive and anti-inflammatory properties [99]. When conjugated to different antigens, CTB induced immune tolerance towards autoantigens in the context of autoimmune diseases or allergies, such as type I diabetes, asthma, Behcet’s disease, atherosclerosis, or Crohn’s disease [18]. These immunosuppressive properties of CT were shown to rely on several different mechanisms: modulation of cytokine production, mucosal generation of regulatory T cells, and induction of tolerogenic antigen-presenting cells and B cells [100]. For example, a treatment for type I diabetes using a CTB-GAD (glutamic acid decarboxylase) fusion protein was shown to suppress the activation of human umbilical cord blood dendritic cells through the down-regulation of pro-inflammatory cytokines (IL-6 and IL-12) and up-regulation of immunosuppressive cytokine IL-10 [101]. Similarly, Denes et al. used a recombinant vaccinia virus rVV-CTB-GAD to treat non-obese diabetic (NOD) mice, which showed a significant decrease in hyperglycemia and pancreatic β islet inflammation [102]. This underlines the potential of CT in antigen uptake and its role in the modulation of the immune response.

Another very interesting therapeutic approach provided by CT consists of targeting and temporarily occupying the ERAD pathway, thus rescuing deleterious phenotypes in genetic diseases with mutations that lead to the premature degradation of a misfolded protein. Adnan et al. illustrated this strategy using inactivated Shiga toxin and CT in cells derived from patients with an F508 deletion in cystic fibrosis transmembrane conductance regulator (CFTR) bronchiolar epithelia, a mutation in a plasma membrane chloride channel that leads to cystic fibrosis [36]. The inactivated toxins were able to induce 5–10-fold increases in protein levels, 20-fold increases in cell surface expression, and 2-fold chloride transport through the membrane with no apparent cytotoxicity. Similarly, they were also able to increase glucocerebrocidase (GCC) by 3-fold in N370SGCC Gaucher’s disease cells, the mutation of which leads to the accumulation of glucocerebrosides in lysosomes. An advantage of this strategy over the use of ERAD inhibitors is that inactivated CT doesn’t induce any ER stress and unfolded protein response (UPR), which can lead to apoptosis. Using a relatively similar approach, Royal et al. designed a CTB subunit with a KDEL ER-retention motif that would induce an UPR response [60]. This UPR response led to TGF-β secretion and increased the wound healing response in vitro in Caco2 cells and in colon explants from patients with inflammatory bowel disease as well as in vivo in a dextran sodium sulfate-induced acute colitis mouse model. These two examples strongly illustrate the potential for hijacking the CT membrane translocation mechanism or its ability to trigger ER stress to treat diseases based on genetic protein misfolding and for mucosal healing in intestinal inflammatory diseases.

In addition to these therapeutic strategies, CT has interesting potential for the treatment of neurological disorders due to its ability to cross the blood-brain barrier (BBB) and internalize into neuronal cells. It has been shown to be particularly efficient in the treatment of glioblastoma in mice [61]. CTB subunits conjugated with paclitaxel-loaded nanoparticles induced apoptosis of intracranial glioma cells and suppressed neovasculature in vivo. Furthermore, this ability of CT to enter neuronal cells has been exploited to develop new neural imaging techniques. Once internalized, the toxin is able to reach the cell body and its dendrites via retrograde transport, which makes it useful for nerve visualization and potentially drug delivery. For example, CTB was conjugated to fluorescent gold nanodots and injected in the sciatic nerve of rats [37]. After 2 days, the fluorescent signal was visible in the spinal cord and was stable for 10 days. This tool could bring an interesting novel visualization technique for the detection of neuronal lesions, further supporting the potential of CT in the development of therapeutic tools.

## 5. Discussion

**Figure 2:**
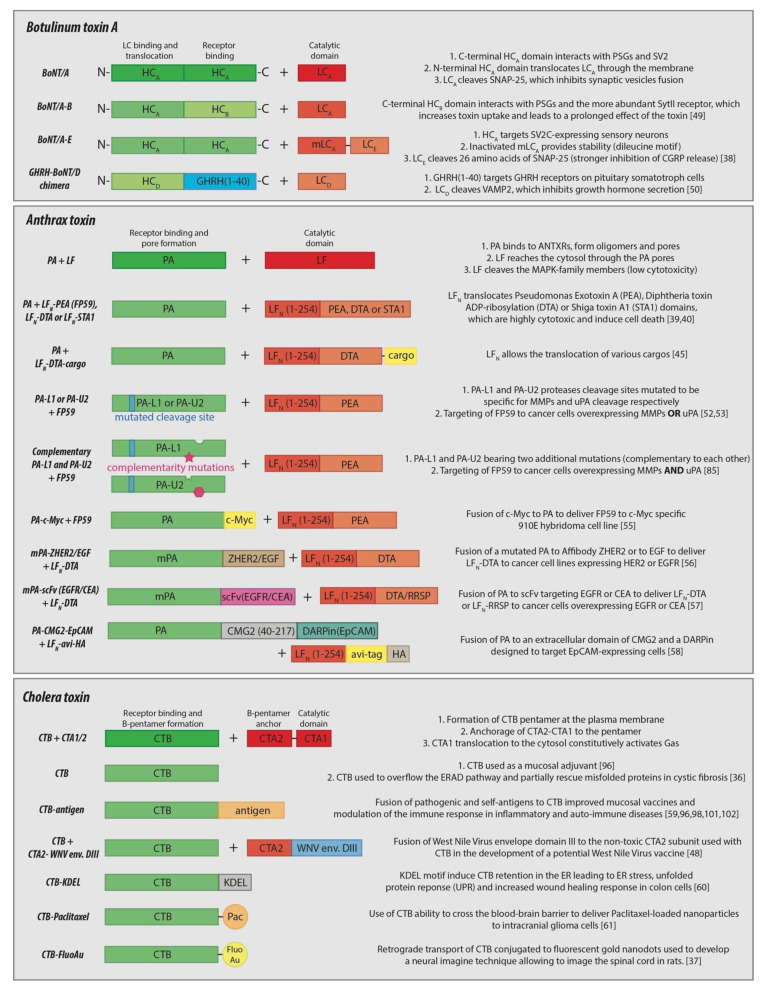
Schematic representation of the different constructs described in this study and brief description of their properties. The three original toxins at the top of their respective compartments are highlighted. A and B domains of each toxin’s subunits are represented in red and green, respectively. The text on the right briefly depict either the internalization process of the original toxin or the therapeutic properties of the chimeric constructs.

In this review, we have illustrated the outstanding diversity of therapeutical strategies provided by the use of botulinum toxin type A, anthrax toxin, and cholera toxin. In addition to the intrinsic therapeutic properties provided by these AB toxins, their modularity in terms of receptor recognition, protease specificity, and non-native cargo delivery allowed the development of many treatments (Figure 2). While the intrinsic properties alone of the three toxins could be therapeutic against specific diseases, their huge potential lies in the possibility of modifying both the A and B subunits of the toxins. The A subunit allows the internalization of non-native cargos into different cell types and in vivo, while the B subunit allows targeting of different receptors and cell types. Several groups have even modulated both subunits of these toxins to deliver drugs or proteins to cells expressing specific non-native receptors, showing the potential of AB toxins as intracellular delivery systems. However, some challenges linked to the immunogenicity and toxicity of these toxins remain to be addressed.

As exogenous proteins, toxins often induce the production of neutralizing antibodies that can interfere with treatments, especially for repeated injections of a drug over a long period of time, like with autoinflammatory diseases [103,104]. In this context, due to its low immunogenicity and, likely also, to the localized mode of administration, BoNT/A triggers the production of neutralizing antibodies in only up to 3% of patients [105]. However, both CT and anthrax toxin were shown to induce the formation of neutralizing antibodies, potentially decreasing the efficiency of an associated drug in long-term therapies [103,104]. This issue could be addressed by investigating potential mutations in the antigens or by using immunosuppressive drugs to decrease the production of neutralizing antibodies [106,107]. In this context, Liu et al. used a combination of cyclophosphamide and pentostatin, two drugs to prevent host-versus-graft rejections, to successfully suppress the antibody production induced by an anthrax-based cancer treatment in mice [87]. However, the risk and benefits have to be carefully weighed when attempting to deliver these therapies together.

Another issue linked to the use of toxins in therapy would be toxicity. While BoNT/A and cholera toxin B are not toxic when used properly, anthrax toxin PA might have a toxic effect as illustrated by its potential role in the Gulf War Illness, in which multi-systemic disease was first observed in Gulf War veterans vaccinated with the PA-based anthrax vaccine [108]. However, this observation needs further validation, as many other chemical or biological factors might have played a role in the development of the disease.

The three bacterial toxins reviewed here have interesting modular properties that could allow their development into various elegant therapeutic strategies. Overall, these toxins have shown new potential therapeutic alternatives in autoimmune and inflammatory diseases, cancer, genetic protein misfolding diseases, movement disorders, and in vaccine development. Although many examples used these three highlighted toxins, several other AB toxins have been shown to have similar characteristics in therapy, such as Shiga toxin and diphtheria toxin, further widening the range of therapeutic possibilities [109,110]. For example, these toxins target different cell types depending on the expression of their receptor. Such specificity can be hijacked to deliver drugs or non-native proteins conjugated to AB toxins to very specific targets in the human organism, as long as the cargo can unfold or is flexible enough to be translocated across the membrane by the B subunit. In addition, one can imagine various ways to target non-native receptors using fusion constructs of the B subunit of AB toxins with Affibodies, DARPins or the natural ligand of the targeted receptor, among others. As described for botulinum toxin and for anthrax toxin in the previous chapters, this elegant strategy has shown promising results and allows for the delivery of cargos to several different cell types with high specificity. Importantly, such systems provide new solutions for the delivery of proteins and peptides which are unable to efficiently translocate through membranes, thereby potentially further increasing the number of new biologics on the market in the coming years.

## Figures and Tables

**Table 1:** Summary of the internalization mechanism steps of botulinum toxin type A, anthrax toxin, and cholera toxin as well as their use in therapeutic applications.

	Botulinum Toxin Type A	Anthrax Toxin	Cholera Toxin
Bacterium	*Clostridium* bacteria family	*Bacillus anthracis*	*Vibrio cholerae*
A subunit	BoNT light chain (LC)	Lethal factor (LF)	Cholera toxin A1 and A2 (CTA1 and CTA2)
B subunit	BoNT heavy chain (HC)	Protective Antigen (PA)	Cholera toxin B (CTB)
Receptors	Polysialogangliosides and SV2 [10,11]	CMG2 and TEM8 [12,13]	GM1 [14]
Oligomerization	None	A_3_B_7_ or A_4_B_8_ [15,16]	AB_5_ [5,17,18]
Cellular compartments	From synaptic membrane to synaptic vesicles [19,20]	From plasma membrane to early endosomes and late endosomes [21]	From plasma membrane to early endosomes and retro-translocation to the Golgi and ER [22,23]
Membrane translocation mechanism	HC translocates LC through the membrane [24]	PA pore translocating LF across the membrane [25,26]	Uses the ERAD-associated translocation mechanism [27]
Cytosolic target	SNAP-25 (of the SNARE complex) [28]	LF cleaves MAPKK family members [29]	Activates Gαs [27]
Therapeutic applications			
Intrinsic properties	Use in the treatment of dystonic and spastic disorders [30] Use in the treatment of autonomic disorders [31] Use in pain therapy [7]	Treatment of cancer relying on the MAPKK-associated pathways [32,33]	Use as adjuvant in mucosal vaccines [34,35] Use of an inactive CT to temporarily occupy the ERAD-associated translocation machinery in the treatments of genetic protein misfolding diseases [36] Use of CTB retro-translocation property to image neuronal cells [37]
A subunit modularity	Cytosolic transport of LC_E_ using LC_A_-HC_A_ as a delivery system [38]	Delivery of non-native protein or small molecule cargos using PA-LF_N_ delivery system [39,40,41,42,43,44,45]	Vaccines development consisting of antigens fused with CTA2 and delivered using CTB [46,47,48]
B subunit modularity	Use of other botulinum toxin HC (i.e HC_B_) to increase LC_A_ uptake [49] Targeting of non-native receptors to inhibit protein secretion [50,51]	Cell-specific delivery of cargos using non-native protease-specific PA [52,53,54] Targeting to non-native receptors using Affibodies, DARPins or receptors ligand for the cytosolic delivery of non-native cargos [55,56,57,58]	Conjugation of self and non-self antigens to CTB to increase antigen uptake in the development of therapies against pathogens, autoimmune and inflammatory diseases [18,59] CTB-KDEL fusion protein to induce ER retention and UPR-mediated wound healing response in colon [60] Use of CTB ability to cross the blood-brain barrier and target neuronal cells to deliver drugs [61]
